# The roles of zinc and copper sensing in fungal pathogenesis

**DOI:** 10.1016/j.mib.2016.05.013

**Published:** 2016-08

**Authors:** Elizabeth R Ballou, Duncan Wilson

**Affiliations:** Aberdeen Fungal Group, MRC Centre for Medical Mycology, School of Medicine, Medical Sciences and Nutrition, University of Aberdeen, Institute of Medical Sciences, Aberdeen, UK

## Abstract

All organisms must secure essential trace nutrients, including iron, zinc, manganese and copper for survival and proliferation. However, these very nutrients are also highly toxic if present at elevated levels. Mammalian immunity has harnessed both the essentiality and toxicity of micronutrients to defend against microbial invasion — processes known collectively as ‘nutritional immunity’. Therefore, pathogenic microbes must possess highly effective micronutrient assimilation and detoxification mechanisms to survive and proliferate within the infected host. In this review we compare and contrast the micronutrient homeostatic mechanisms of *Cryptococcus* and *Candida* — yeasts which, despite ancient evolutionary divergence, account for over a million life-threatening infections per year. We focus on two emerging arenas within the host–pathogen battle for essential trace metals: adaptive responses to zinc limitation and copper availability.

## Highlights

• Zinc and copper are essential trace elements required for cell function.
• Nutrient Immunity restricts zinc and copper access and mediates toxicity.
• Divergent fungi integrate zinc and copper responsive regulons for pathogenesis.

**Current Opinion in Microbiology** 2016, **32**:128–134

This review comes from a themed issue on **Host-microbe interactions: fungi**

Edited by **Elaine Bignell** and **Bart PHJ Thomma**

For a complete overview see the Issue and the Editorial

Available online 18th June 2016

**http://dx.doi.org/10.1016/j.mib.2016.05.013**

1369-5274/© 2016 The Author(s). Published by Elsevier Ltd. This is an open access article under the CC BY license (http://creativecommons.org/licenses/by/4.0/).

## Introduction

The concept of nutritional immunity, in terms of host-driven iron sequestration, has been appreciated for decades: our bodies maintain extremely low iron cation levels via intracellular sequestration (ferritin, haemoglobin, the hepcidin axis) and expression of extracellular high-affinity iron-binding proteins (transferrin, lactoferrin). Successful pathogens have in turn evolved effective assimilation mechanisms (high-affinity transporters, siderophores and specialised binding proteins) [1]. However, additional layers of nutritional immunity have been revealed in recent years: micronutrient restriction is not limited to iron, with host zinc and manganese sequestration playing key roles in controlling infection [2]. Moreover, in certain host niches, pathogens can be exposed to potentially toxic levels of iron, zinc and copper. Therefore, beyond high-affinity iron uptake systems, microbial pathogens must also possess highly effective homeostatic mechanisms (both assimilation and detoxification) for other trace metals within their hosts [2].

Host nutritional immunity during fungal infection and the pathogen assimilation pathways employed in counterattack have been extensively discussed in a number of recent reviews [3, 4, 5, 6]. In this mini-review we focus on two emerging areas: the adaptive responses of *Candida* and *Cryptococcus* species to changes in environmental zinc and copper.

## Divergent virulence factor intercalation of the zinc regulon

Zinc is the second most abundant transition metal in the human body but, like iron, its availability to microbial pathogens is strictly limited. Zinc restriction is further compounded during periods of inflammation due to the action of calprotectin. Calprotectin constitutes approximately half the cytoplasmic protein content of neutrophils, is a dominant component of NETs (*n*eutrophil *e*xtracellular *t*raps) and elicits antifungal activity via zinc chelation [7]. Calprotectin is a heterodimer of S100A8 and S100A9, members of the S100 family of low molecular weight proteins. Antifungal activity of another S100 family member, psoriasin (S100A7) has recently been reported to elicit antifungal activity via zinc-sequestration [8^•^], suggesting that multiple members of this protein family may have similar functions. Furthermore, host cell internalisation and subcellular compartmentalisation further limit accessibility to extracellular and intracellular pathogens, respectively. For example, activated, *Histoplasma*-infected macrophages shuttle zinc to their Golgi, restricting fungal proliferation and promoting clearance [9].

Interestingly, contemporary pathogenic fungal species appear to simultaneously exhibit remarkably similar and divergent responses to changes in zinc availability. First described in the model yeast *Saccharomyces cerevisiae*, Zap1 is a zinc finger transcription factor that regulates the expression of zinc transporter-encoding genes and is essential for maintaining zinc homeostasis. Under zinc limitation, Zap1 exhibits positive auto-regulation, rapidly amplifying its own transcription and subsequent protein levels and positively regulating the expression of zinc transporter-encoding genes, including *ZRT1*, *ZRT2* and *ZRT3*. For a detailed discussion of *S. cerevisiae* Zap1 function, readers are directed to an excellent recent review by Wilson and Bird [10].

**Figure 1:**
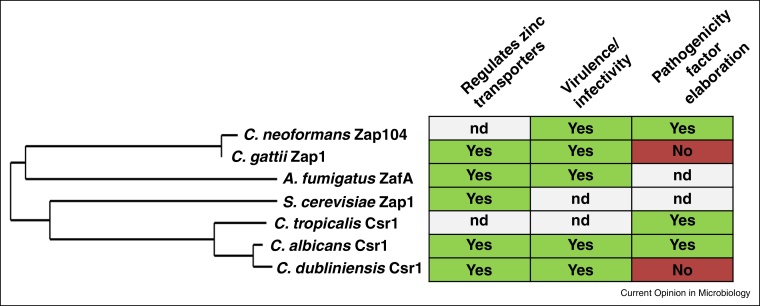
Phylogenetic and functional relationships of Zap1 orthologues in fungi. The predicted amino acid sequences of Zap1 orthologues were downloaded from FungiDB, *Aspergillus* Genome Database, *Saccharomyces* Genome Database and *Candida* Genome Database and aligned using Phylogeny.fr. Note that in all reported cases, Zap1 regulates the expression of zinc transporters and is required for virulence or infectivity in animal models of infection, but that confirmed pathogenicity factor expression is species-specific.

It would appear that Zap1 orthologues function as conserved master regulators of zinc homeostasis in fungi: in the pathogenic fungal species *Candida albicans*, *Candida dubliniensis*, *Aspergillus fumigatus*, and *Cryptococcus deuterogattii*, Zap1 orthologues (also known as Csr1 in *Candida*, ZafA in *Aspergillus*) have been directly demonstrated to regulate the expression of zinc transporter-encoding genes. Given the evolutionary divergence of these species, it is plausible that orthologues of Zap1 regulate zinc homeostasis in many (if not all) fungi [11, 12•, 13, 14]. Underscoring the importance of fungal zinc homeostasis during infection, each fungal pathogen tested to date exhibits virulence attenuation or decreased infectivity upon deletion of their respective *ZAP1* orthologue in relevant animal models of infection (Figure 1).

Despite this apparent conservation of function as a regulator of zinc homeostasis and virulence, there may be differences in the Zap1-regulons of different contemporary fungal species. *Cryptococcus neoformans* and *C. deuterogattii* are closely related species that express three major virulence factors for infectivity: capsule formation, melanin production and growth capacity at 37 °C. Interestingly, while *C. deuterogattii ZAP1* is dispensable for capsule and melanin production [14], *C. neoformans* cells lacking the *ZAP1* orthologue (*ZAP104*) are deficient in capsule, melanin, and a/α cell fusion, suggesting rewiring [15, 16•]. While we cannot rule out that *C. deuterogattii* Zap1 and zinc homeostasis may influence melanin and capsule formation under conditions not examined by Schneider *et al.* [14], Liu *et al*. and Jung *et al*. independently demonstrate *ZAP104* involvement in virulence factor expression (melanin and capsule synthesis) in *C. neoformans* [14, 15, 16•]. These three studies together suggest that Zap1-mediated zinc homeostasis in *C. neoformans*, but not *C. deuterogattii*, may impinge on virulence factor (capsule/melanin) expression, and raise questions about the reasons for phenotypic differences. An examination of Zap1 and Zap104 protein sequences immediately reveals structural differences: where the Zap1 N-terminus encodes a single zinc-binding domain, Zap104 encodes two [14]. Whether these structural differences lead to differing sensitivities to zinc availability or differences in promoter binding remain to be explored.

In the *Candida* genus, Zap1 function has been investigated in three relatively closely related species [17, 18]: *C. albicans*, *C. dubliniensis* and *C. tropicalis*. Despite diverging from basidiomycetes approximately 500 mya [19], *C. albicans*, like *C. neoformans*, appears to regulate major virulence attributes (hyphal morphogenesis, adhesion and biofilm maturation) via Zap1 (note that Zap1 in *Candida* is also known by the common name Csr1). Kim *et al*. [20] first reported that *ZAP1* deletion in *C. albicans* precluded hyphal morphogenesis under a range of environmental conditions. Moreover, overexpression of *ZRT1* or *ZRT2* (encoding the two predicted plasma membrane zinc transporters of *C. albicans*) promoted filamentous growth of the *zap1*Δ mutant on certain media, suggesting that the morphogenic defect may be due to perturbed cellular zinc homeostasis rather than direct Zap1-regulation of hypha-formation.

A recent study of *C. dubliniensis* found that, in this close relative of *C. albicans*, Zap1 also positively regulates *ZRT1*, *ZRT2* and *PRA1* [12^•^], reinforcing the concept that Zap1 is the universal regulator of zinc assimilation in the fungal kingdom (Figure 1). However, unlike *C. albicans*, the *C. dubliniensis zap1*Δ mutant was not defective for hypha formation. On the other hand, Zap1 does regulate filamentation in *C. tropicalis* [21].

Using a powerful combination of large-scale mutant library screening, transcriptional profiling, chromatin immunoprecipitation, regulatory network analysis and infection modelling, the group of Aaron Mitchell found that Zap1 regulates extracellular matrix production by *C. albicans* biofilms and governs the expression of adhesin molecules. The *zap1*Δ mutant efficiently formed biofilms; however, these structures produced highly elevated levels of β-glucan, indicating that Zap1 negatively regulates extracellular matrix production — a key attribute in biofilm maturation [11]. Zap1 occupies *ZRT1*, *ZRT2* and *PRA1* promoters and positively regulates their transcription, in agreement with Zap1 acting as the master regulator of zinc homeostasis in *C. albicans*. However, overexpression of these zinc assimilation factors in the *zap1*Δ mutant had no effect on β-glucan production, suggesting that cellular zinc homeostasis *per se* may not directly regulate extracellular matrix synthesis, and that Zap1 possesses additional regulatory function [11]. Indeed, Zap1 was also found to be required for the regulation of genes involved in adhesion — another key element of biofilm formation [22]. Despite this regulatory role, the *zap1*Δ mutant itself exhibited wild type levels of adhesion. Therefore, to test whether Zap1 functions redundantly in adherence regulation, these authors tested the effect of *ZAP1* overexpression in strains lacking other transcription factors important for adhesion. This approach demonstrated that *ZAP1* overexpression restored the adhesive capacity of multiple adhesion-defective transcription factor mutants. This was most likely due to Zap1-dependent transcriptional induction of CSTAR (*c*ell *s*urface *t*argets of *a*dherence *r*egulators) genes, as *ZAP1* overexpression was found to increase the expression of these target genes in mutants lacking three direct regulators of adhesion (*zcf28*Δ, *try2*Δ and *try3*Δ). Therefore, in addition to zinc assimilation, *C. albicans* appears to have intercalated key virulence attributes — adhesion and biofilm maturation — into its Zap1 regulon. It should be noted that, in these studies, zinc limited media was not used, and Zap1 may regulate different subsets of genes, depending on the environmental zinc status.

In summary, it would appear that beyond zinc homeostasis, the Zap1 transcription factor plays different roles in different fungal species and, at least in *C. neoformans* and *C. albicans*, these are directly associated with the expression of ‘classical’ pathogenicity factors. So why might fungal pathogens place control of key virulence attributes within their Zap1 regulon? One likely possibility is the relative zinc levels found in the natural reservoirs of these yeasts, compared to the infected host. Both arboreal environments and the mammalian digestive tract are relatively high zinc environments, whilst nutritional immunity creates extreme zinc depletion within infected tissues. Hardwiring virulence factor expression into their zinc starvation-responses may contribute to the high pathogenic potential of these species, similar to the situation for iron [23].

## Dynamic regulation of response to nutritional copper immunity

In contrast to zinc nutritional immunity, which functions primarily via micronutrient sequestration by the host, copper nutritional immunity appears to represent a highly dynamic system [3, 23]. During *C. albicans* bloodstream infection, serum copper levels rise early in infection [24]. Upon kidney colonisation, an early spike in copper levels is followed by rapid copper sequestration. For *C. neoformans*, while the lungs are a high copper environment, alveolar macrophages induce host *CTR1* but repress the *ATP7A* transporter for phagosomal copper compartmentalization, resulting in copper starvation upon engulfment [25]. Consistent with this, *C. neoformans* requires the copper transporter *CTR4* for survival within alveolar macrophages but it is dispensable for growth in the lungs [26••, 27]. Although cerebral spinal fluid (CSF) copper levels are estimated at 100 μM under certain conditions, the CSF is generally copper poor and induces copper transporter expression [27, 28]. Likewise, upon crossing the blood–brain-barrier, the low copper availability of the brain necessitates *CTR1* or *CTR4* for *C. neoformans* virulence [27]. Responding to this dynamic environment requires a coordinated transcriptional response on the part of the fungus.

**Figure 2:**
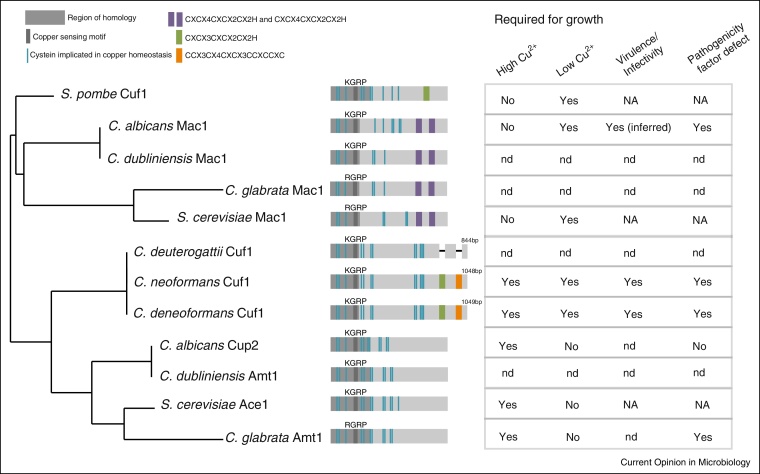
Phylogenetic and structural relationships of copper responsive transcription factors in fungi. The predicted amino acid sequences of the indicated orthologs were downloaded from FungiDB, Broad, NCBI, *Saccharomyces* Genome Database, Pombase, and *Candida* Genome Database and aligned using Phylogeny.fr. Structural schematics were constructed based on sequence analysis and functional analysis is based on the reported literature, discussed in the text.

Structurally, fungal copper responsive transcription factors fall into three classes typified by *S. cerevisiae* Ace1 and Mac1 and *S. pombe* Cuf1 (Figure 2). All maintain an N-terminal copper responsive (R/K)GRP motif and conserved N-terminus [29^•^]. In ScAce1 and ScMac1 residues 1–40 encode a zinc-binding domain. ScAce1 residues 40–110 encode CXC and CX_2_C motifs necessary to coordinate a tetra-copper cluster for binding MRE promoter motifs. Mac1 is characterised instead by dual cysteine rich C-terminal motifs, REP-I (CXCX_4_CXCX_2_CX_2_H) and REP-II (CXCX_4_CXCX_2_CX_2_H), which sense copper and stabilise DNA binding, respectively[30, 31]. Specificity is achieved through division of labour for high and low copper responses: ScMac1 regulates the expression of the *CTR1* and *CTR2* copper importers in response to copper limitation, while copper toxicity triggers Mac1 degradation and assembly of the ScAce1 tetra-copper cluster, activating DNA-binding and induction of *CUP1* and *CRS5* metallothioneins and *SOD1*, which in turn further negatively regulates Mac1 [32, 33, 34]. Likewise, the *C. glabrata* Ace1 orthologue Amt1 is required for high copper and metallothionein expression and *C. glabrata* encodes a putative Mac1 homolog with overall structural similarity to ScMac1 [35, 36] (Figure 2).

SpCuf1 is representative of the third class, with a single, structurally distinct REP motif: CXCX_3_CXCX_2_CX_2_H. SpCuf1 is required for growth on limited copper through binding CuSE motifs in the *CTR4* promoter; in the presence of high copper, Cu-REP interaction induces a conformational change masking the N-terminal nuclear localisation signal and inhibiting function [37]. *S. pombe* lacks metallothineins, but detoxifies copper through a dual function SOD chaperone, PCCS [38].

*C. albicans* responds to both low and high copper levels via CaMac1, which induces either Cu-*SOD1* in high copper or Mn-*SOD3* in low copper, along with the *CTR1* copper importer; both Ca*MAC1* and *CTR1* are required for hyphal growth, and *SOD1* is required for virulence [24, 39, 40, 41]. *CaACE1/CUP2* is additionally required for growth on high copper and regulates *CUP1* and *CRD2* metallothioneins [42, 43]. *C. dubliniensis* does encode *MAC1* and *AMT1* homologues that presumably act in a similar fashion, however their targets remain uncharacterised (Figure 2).

*Cryptococcus* Cuf1 is required for growth on both low and high copper and is structurally distinct from SpCuf1 and ScMac1/Ace1: in addition to the Cuf1 REP motif, a second cysteine-rich repeat (CCX3CX4CXCX3CCXCCXC) is conserved in both *C. neoformans* and *C. deneoformans*, estimated to have diverged 18–24 mya [44, 45]. For the outbreak strain *C. deuterogattii*, Cuf1 sequences in multiple isolates (R265, LA55, RAM5) have undergone truncations that specifically excise the copper responsive REP motifs (Figure 2), although these motifs are maintained in *C. decagattii* (*IND107*), *C. gattii* (EJB2, Ru294, WM276) and *C. bacillisporus* (CA1873, Ca1280). The relationship between copper and Cuf1 activity has not yet been reported in these organisms.

For both *C. neoformans* and *C. deneoformans*, growth in low copper requires *CTR1/CTR2* (*CNAG_07701*) and *CTR4* [25, 26••, 46, 47]. While *CTR1* is constitutively expressed, *CTR4* is specifically induced by low copper in a *CUF1*-dependent manner. During growth on high copper, *CUF1* mediates *CMT1* and *CMT2* metallothionein expression [46]. Similarly, Lin *et al*. report that *C. deneoformans* Mac1/Cuf1 is required for growth on both high and low copper [48]. This dual activity represents a rewiring in comparison to Cuf1 activity in *S. pombe*, where high copper leads to the sequestration of Cuf1 in the cytoplasm [37]. In addition to growth, copper levels directly influence *Cryptococcus* virulence through capsule and melanin via Cuf1-independent and Cuf1-dependent mechanisms. In both species, Cuf1 is required for melanin production in a low copper environment, likely because melanin synthesis requires the *LAC1* copper-dependent oxidase and a *cuf1Δ* mutant is unable to acquire sufficient copper from the environment [46, 48, 49, 50]. Lac1 copper loading is mediated by the *CCC2* P-type copper transporting ATPase, *ATX1* copper chaperone, and the *ClC-A* chloride channel. However, in copper-replete conditions, Cn*cuf1Δ* melanin defects persist in the absence of growth defects [16•, 47], and the *cuf1Δ* null is more readily phagocytosed [51]. Although a complete transcriptional analysis of Cuf1 and copper homeostasis during virulence factor induction has not been performed, *CTR1* and *CTR4*, but not *CUF1*, are induced by the temperature shift sufficient to induce capsule during growth in DMEM [51, 52]. In *C. deneoformans*, excess copper induces filamentation and is dependent on Ccc2 activity [48]. Interestingly, a *C. albicans gpa2Δ/Δ* mutant is deficient for *CTR1* and *FRE7* copper importer expression and inappropriately expresses *CRD2*, suggesting a role for cAMP/PKA in copper homeostasis and hyphal growth. In *C. neoformans*, cAMP/PKA regulates melanin and capsule via integration with the pH-responsive transcription factor Rim101 and ESCRT [53, 54]. Cn*rim101* and ESCRT mutants vps25 and rim20 are sensitive to low copper, although capsule and immune evasion defects in these mutants are likely due to changes in the cell wall rather than defects in expression [51, 55]. *CnCCC2* and *ATX1* are also required for growth on low iron, likely due to the interaction between copper and iron uptake [49].

In summary, *Candida* and *Cryptococcus* species experience significant shifts in zinc and copper availability upon transitions between commensal or environmental and infective stages. It would appear that certain successful fungal pathogens have hardwired virulence factor expression into their metal ion sensing machinery and may use the metal ion environment of the host as a key signal. A major challenge for the future will be to understand how these signals are integrated, and whether we can therapeutically target these pathways to treat fungal infections.

## References and recommended reading

Papers of particular interest, published within the period of review, have been highlighted as:

• of special interest
•• of outstanding interest
